# Wnt-*β*-Catenin Signaling Promotes the Maturation of Mast Cells

**DOI:** 10.1155/2016/2048987

**Published:** 2016-10-23

**Authors:** Tomoko Yamaguchi, Misae Nishijima, Katsuhisa Tashiro, Kenji Kawabata

**Affiliations:** ^1^Laboratory of Stem Cell Regulation, National Institutes of Biomedical Innovation, Health and Nutrition, Saito-Asagi 7-6-8, Ibaraki, Osaka 567-0085, Japan; ^2^Laboratory of Biomedical Innovation, Graduate School of Pharmaceutical Sciences, Osaka University, Yamadaoka 1-6, Suita, Osaka 565-0871, Japan

## Abstract

Mast cells play an important role in the pathogenesis of allergic diseases. Immature mast cells migrate into peripheral tissues from the bone marrow and undergo complete maturation. Interestingly, mast cells have characteristics similar to hematopoietic stem cells (HSCs), such as self-renewal and c-kit expression. In HSCs, Wnt signaling is involved in their maintenance and differentiation. On the other hand, the relation between Wnt signaling and mast cell differentiation is poorly understood. To study whether Wnt signals play a role in the maturation of mast cells, we studied the effect of Wnt proteins on mast cell maturation of bone marrow-derived mast cells (BMMCs). The expression levels of CD81 protein and histidine decarboxylase mRNA and activity of mast cell-specific protease were all elevated in BMMCs treated with Wnt5a. In addition, Wnt5a induced the expression of Axin2 and TCF mRNA in BMMCs. These results showed that Wnt5a could promote the maturation of mast cells via the canonical Wnt signaling pathway and provide important insights into the molecular mechanisms underlying the differentiation of mast cells.

## 1. Introduction

Mast cells reside in perivascular regions in many tissues and regulate both innate and adaptive immunity [1]. Mast cells, which are derived from hematopoietic stem cells (HSCs), migrate into peripheral tissues including skin and complete their differentiation within the tissue environment [2, 3]. Bone marrow-derived mast cells (BMMCs), which can be obtained by prolonged culture of bone marrow cells in the presence of IL-3, have been widely used as a standard culture model for mast cells, because they represent several aspects of mast cells, such as surface expression of Fc*ε*RI and c-kit, and histamine release upon antigen stimulation. However, as BMMCs are slightly immature and are incapable of mimicking the specific features of cutaneous mast cells, such as degranulation upon exposure to an IgE-independent stimulus, novel culture models using more mature mast cells are required for mimicking cutaneous mast cells. Previous studies showed that coculture of BMMCs with Swiss 3T3 fibroblasts in the presence of stem cell factor (SCF) facilitated morphological and functional maturation toward a connective tissue-type mast cell- (CTMC-) like phenotype [4], although the maturation mechanism of mast cells is still poorly understood.

Wnt signaling controls a variety of developmental processes such as proliferation, migration, and differentiation [5, 6]. Both Wnt ligands and their receptors, Frizzleds (Fzds), form multigene families, with a large number of possible ligand-receptor interactions. Wnt signaling pathways have been divided into two broad categories: (1) the canonical pathway, which is dependent on the stabilization of *β*-catenin and its translocation to the nucleus by Wnt ligands including Wnt3a, and (2) the noncanonical pathways, which comprise all *β*-catenin-independent Wnt-induced signaling events by Wnt ligands including Wnt5a [7, 8]. Recently, Wnts have been shown to induce inflammatory genes such as interleukins and matrix metalloproteinases (MMPs), suggesting that Wnt signaling is implicated in inflammatory regulation [9, 10]. The specific role of mast cells in inflammatory disease is still unclear, although some groups reported that mast cells are involved in the initiation of rheumatoid arthritis (RA) and experimental autoimmune encephalomyelitis (EAE) [11, 12]. However, the relation between Wnt signaling and maturation of mast cells is still poorly understood. Moreover, interestingly, mast cells have characteristics similar to HSCs, such as self-renewal ability and c-kit expression. In HSCs, Wnt signaling is involved in their maintenance [13, 14].

In this study, we investigated the role of Wnt signaling during the terminal differentiation of mast cells. Our results showed that Wnt5a promoted the terminal differentiation of mast cells via the canonical Wnt pathway.

## 2. Materials and Methods

### 2.1. Generation of BMMCs

C57BL/6 mice were purchased from Nippon SLC, and all animals were maintained under specific pathogen-free conditions. Bone marrow cells were prepared from the femurs and tibiae of mice. Cells were cultured in RPMI 1640 medium containing 10% fetal bovine serum (FBS), 1% nonessential amino acids (NEAA), and 10 ng/mL IL-3 (R&D Systems). Culture medium was replaced with fresh medium every 5 days. After 4 weeks of culture, we confirmed the cell surface expression of both Fc*ε*RI and c-kit (>98% positive). The protocol was approved by the Animal Care and Use Committee of the National Institute of Biomedical Innovation, Health and Nutrition (permit number: DS19-106R14) in accordance with their guidelines. Daily feeding/watering were done* ad libitum*. Mice were euthanized by cervical dislocation under anesthesia and used for experiments.

### 2.2. Cell Culture

OP9 stromal cells were cultured in *α*-minimum essential medium (*α*-MEM: Sigma) supplemented with 20% FBS, 2 mM L-glutamine (Invitrogen), and 1x NEAA. Swiss 3T3 fibroblast was kindly provided by Dr. Tanaka (Okayama University) [4]. Swiss 3T3 fibroblasts were cultured in RPMI-1640 medium (Sigma) supplemented with 10% FBS, 1x NEAA, and antibiotics.

### 2.3. Purification of Peritoneal Mast Cells

Peritoneal mast cells were purified from total peritoneal cells. Cells were incubated with anti-dinitrophenyl (DNP) IgE (SPE7, Sigma-Aldrich) for 1 h. After washing with FACS buffer (PBS/2% FBS), cells were stained with fluorescence isothiocyanate- (FITC-) conjugated monoclonal anti-mouse IgE (RME-1, BioLegend) and phycoerythrin- (PE-) conjugated monoclonal anti-mouse c-kit (ACK2, eBioscience) antibodies in the presence of anti-CD16/32 antibody to block the nonspecific binding. IgE/c-kit-double positive cells were isolated by an SH-800 (Sony) cell sorter. Dead cells were excluded from the analysis by staining with 4′,6-diamidino-2-phenylindole (DAPI: Sigma).

### 2.4. Reverse Transcription and Quantitative Polymerase Chain Reaction (RT-PCR)

Total RNA was isolated from mast cells by using RNAiso Plus reagent (Takara) and NucleoSpin RNAXS (Takara). The cDNA was synthesized using SuperScript VILO cDNA synthesis kit (Life Technologies), and semiquantitative PCR was then performed using TAKARA Ex Taq HS DNA polymerase (Takara). The level of HDC, MCP5, CPA, Axin2, and Tcf7 mRNA was quantified using the SYBR Green detection system (Applied Biosystems). Results were normalized by expression of the GAPDH transcripts. The sequences of the primers used in this study are listed in Tables 1 and 2.

### 2.5. Flow Cytometry

Nonadherent cells were harvested from the culture plate and washed with ice-cold PBS by centrifugation. Cell suspensions were stained with FITC-conjugated monoclonal anti-mouse Fc*ε*RI (MAR-1, eBioscience), APC-conjugated monoclonal anti-mouse c-kit, and PE-conjugated monoclonal anti-mouse/rat CD81 (Eat-2, BioLegend) antibodies in the presence of anti-CD16/32 antibody. The stained cells were washed and resuspended in 1% FBS-PBS, and data acquisition was performed on LSRFortessa (BD Biosciences) and data were analyzed with FACS Diva software (BD Biosciences).

### 2.6. Protease Assay

Tryptase and carboxypeptidase A (CPA) activities were measured as described previously [15]. BMMCs were washed with PBS, lysed in PBS containing 2 M NaCl/0.5% Triton X-100, and incubated on ice for 30 min. The lysate was centrifuged at 12,000 rpm for 30 min at 4°C. The activities of granule proteases in the resultant supernatants were measured using their specific chromogenic peptide substrates, such as S-2288 for tryptase (Sekisui medical) and M-2245 for CPA (Bachem).

### 2.7. *β*-Hexosaminidase Release Assay

*β*-Hexosaminidase activity was measured as a marker of the granular fraction for evaluation of degranulation. Cells were washed with HEPES buffer (137 mM NaCl, 20 mM HEPES, 5 mM D-glucose, 2.7 mM KCl, 0.4 mM NaH_2_PO_4_, 0.5 mM MgCl_2_, 2.4 mM CaCl_2_, and 0.1% BSA) and incubated with the buffer containing compound 48/80 (10 *μ*g/mL, Sigma) for 30 min.

### 2.8. Western Blot Analysis

Cells were homogenized with lysis buffer (20 mM HEPES, 2 mM EGTA, 10% glycerol, 1% Triton X-100, 5 mM DTT, and 2 mM PMSF). After being frozen and thawed, the homogenates were centrifuged at 15,000 ×g for 10 min at 4°C, and the supernatants were collected. The lysates were subjected to SDS-PAGE on 7.5% polyacrylamide gel and were then transferred onto polyvinylidene fluoride membranes (Millipore). After the reaction was blocked with 5% skim milk in TBS containing 0.1% Tween 20 at room temperature for 1 h, the membranes were incubated with mouse anti-*β*-catenin (Santa Cruz Biotechnology), anti-LRP5 (Santa Cruz Biotechnology), anti-Fzd4 (R&D Systems) or mouse anti-*β*-actin (Sigma-Aldrich) antibodies at 4°C overnight, followed by reaction with HRP-conjugated anti-mouse IgG antibody (Cell signaling Technology) at room temperature for 1 h. The bands were visualized by ECL Plus Western blotting detection regents (GE Healthcare) and the signals were read using a LAS-4000 imaging system (FUJI Film).

### 2.9. Coculture of Mast Cells with Swiss 3T3 Fibroblasts

BMMCs were cocultured with mitomycin C-treated Swiss 3T3 fibroblasts in the presence of 100 ng/mL SCF and 1 *μ*g/mL normal rat IgG (R&D Systems) or 1 *μ*g/mL anti-Wnt5a antibody (R&D Systems). The subculture was performed every 4 days. The cells were trypsinized and replated, and nonadherent cells were collected as mast cells and used for further analysis.

### 2.10. Statistical Analysis

Statistical analysis was performed using unpaired two-tailed Student's *t*-test.

## 3. Results

### 3.1. Expression of Wnt Receptors in Mast Cells

**Figure 1:**
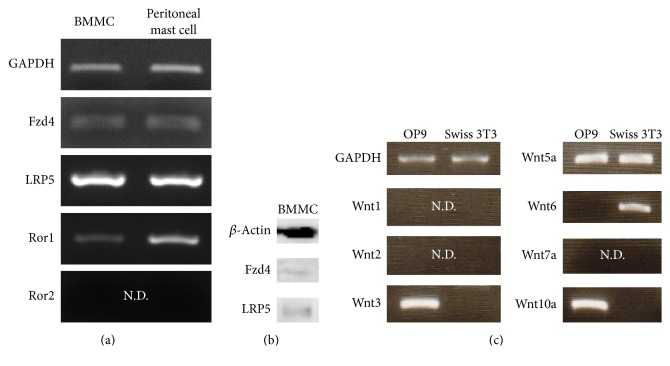
Expression of Wnt receptor (Fzd, Ror, and LRP) and Wnt ligand. (a) The expression of various Wnt receptor mRNAs in mast cells was examined by conventional RT-PCR. (b) The expression of Wnt receptors (Fzd4 and LRP5) in mast cells was detected by Western blot analysis. (c) The expression of Wnts mRNAs in feeder cells was examined by conventional RT-PCR. N.D: not detected.

We studied the expression of Wnt receptors, including Fzd, low-density lipoprotein receptor-related protein (LRP), and receptor tyrosine kinase-like orphan receptor (Ror), on mast cells. Semiquantitative RT-PCR analysis showed that Fzd4 and LRP5 were expressed in both BMMCs and peritoneal mast cells (Figure 1(a)). Ror1, but not Ror2, was expressed in both BMMCs and peritoneal mast cells (Figure 1(a)). In addition, Fzd4 and LRP5 proteins were detected in mast cells by Western blot analysis (Figure 1(b)), indicating the possibility that Wnt/Fzd-mediated signals might regulate the maturation of mast cells.

### 3.2. Expression of Wnt Ligand in OP9 Cells and Swiss 3T3 Fibroblasts

Previously, we reported that Swiss 3T3 fibroblasts and OP9 cells promoted the maturation of mast cells [15]. Therefore, we examined the mRNA expression of Wnt genes in OP9 cells and Swiss 3T3 fibroblasts. Semiquantitative RT-PCR analysis revealed that Wnt5a was expressed in both OP9 cells and Swiss 3T3 fibroblasts (Figure 1(c)). Therefore, we focused on the role of Wnt5a in the maturation of mast cells.

### 3.3. Wnt5a Enhanced Maturation of Mast Cells via the Canonical Pathway

**Figure 2:**
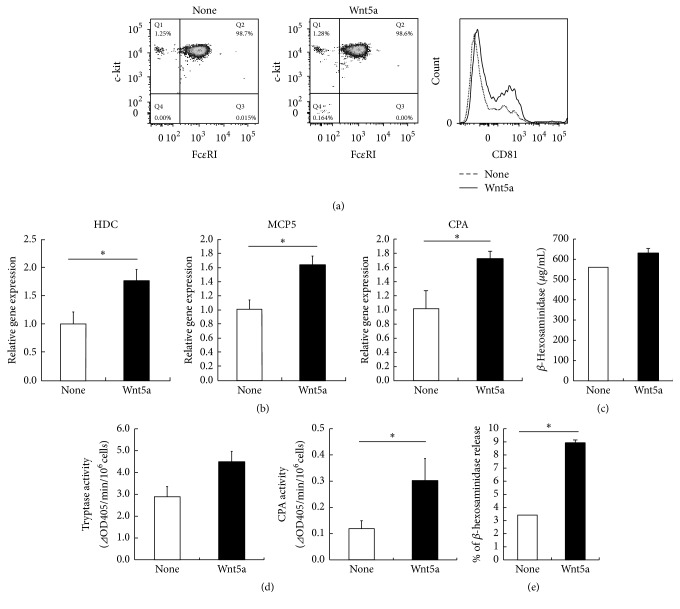
Maturation of mast cells by Wnt5a. (a–e) BMMCs were cultured for 20 days under two conditions: one with IL-3 (1 ng/mL), SCF (100 ng/mL), and Wnt5a (50 ng/mL) and the other with only IL-3 (1 ng/mL) and SCF (100 ng/mL) (control). (a) BMMCs were stained with fluorescence-labeled anti-Fc*ε*RI, anti-c-kit, and anti-CD81 antibodies for 30 min on ice. The stained cells were washed, resuspended in 1% FBS-PBS, and analyzed by flow cytometry. Surface marker expression was analyzed by gating on viable cells in the FSC/SSC. One representative dot plot out of five independent experiments is shown. (b) Gene expression levels of HDC, MCP5, and CPA were measured by quantitative RT-PCR. Gene expression levels in the control were taken as 1.0. (c) *β*-Hexosaminidase content was also measured. (d) Cell extracts prepared from BMMCs were assayed for tryptase and CPA activities as described in Section 2. (e) *β*-Hexosaminidase release from cells after stimulation with compound 48/80. All data represent the means ± SD (*n* = 4). ^*∗*^ *p* < 0.05.

In order to test the functions of Fzds in mast cells, BMMCs were treated with Wnt5a and the surface expression of c-kit and Fc*ε*RI on BMMCs was examined. Wnt5a did not affect the surface expression of either Fc*ε*RI or c-kit on mast cells (Figure 2(a)). CD81 is considered to be a marker of CTMCs [4]. FACS analysis showed that expression of CD81 was elevated in the BMMCs treated with Wnt5a (Figure 2(a)). Histidine decarboxylase (HDC) is a critical enzyme that is involved in the synthesis of endogenous histamine in mammals [16, 17] and is considered to be one of the indices of mast cell maturation [17]. Therefore, we compared the expression of HDC mRNA levels in BMMCs in the presence or absence of Wnt5a and found that Wnt5a induced the expression of HDC mRNA (Figure 2(b)). In addition, the expression levels of MCP5 and CPA mRNA were significantly upregulated in BMMCs in the presence of Wnt5a (Figure 2(b)). Next, we measured the amount of *β*-hexosaminidase, a marker enzyme for degranulation, in BMMCs in the presence or absence of Wnt5a. Our results showed that Wnt5a could increase the amount of *β*-hexosaminidase (Figure 2(c)). These results suggest that Wnt5a could promote the synthesis of histamine. To further compare the degrees of mast cell maturation in the presence or absence of Wnt5a, we measured the tryptase and CPA activities in BMMCs. BMMC treated with Wnt5a showed a substantial increase in the tryptase and CPA activities (Figure 2(d)). As a result of examination of response to compound 48/80, Wnt5a-treated BMMCs showed the significant exocytosis of *β*-hexosaminidase (Figure 2(e)). These results suggest that Wnt5a can promote the maturation of BMMCs into CTMC-like cells.

**Figure 3:**
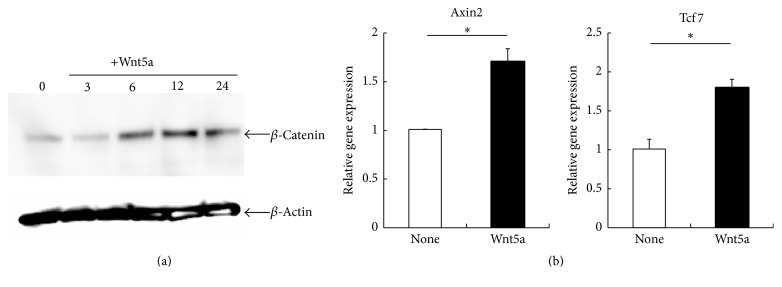
Activation of Wnt canonical signaling in mast cells by Wnt5a. (a) BMMCs were cultured with Wnt5a (50 ng/mL), and then the expression of *β*-catenin in these cells was detected in Western blot analysis. Representative results from one of three independent experiments performed are shown. (b) Gene expression levels of Axin2 and TCF were measured by quantitative RT-PCR. Gene expression levels in the control were taken as 1.0. All data represent the means ± SD (*n* = 4). ^*∗*^ *p* < 0.05.

In general, Wnt5a is known to activate *β*-catenin-independent pathway (noncanonical Wnt pathway). Previously, Mikels and Nusse demonstrated that Wnt5a can also activate the Wnt/*β*-catenin pathway (canonical Wnt pathway) in the presence of the appropriate Wnt receptors, Fzd4 and LRP5, but not in the presence of Ror2 [18]. Semiquantitative RT-PCR showed that Fzd4 and LRP5, but not Ror2, were expressed in mast cells (Figure 1). These results suggest that the canonical Wnt signaling pathway but not the noncanonical Wnt signaling pathway by Wnt5a is implicated in the maturation of mast cells. We next investigated the accumulation of *β*-catenin, a key molecule of the canonical pathway, in the absence or presence of Wnt5a. When treated with Wnt5a for 3 h, *β*-catenin accumulation was observed in whole cell lysates (Figure 3(a)). A previous study demonstrated that Wnt/*β*-catenin signaling induces the transcription of Axin2 and TCF [19]. Therefore, quantitative RT-PCR analysis was performed to compare the expressions of Axin2 and TCF mRNA levels in BMMCs. Our results showed that Wnt5a induced the expression of Axin2 and TCF mRNA (Figure 3(b)). These results suggest that Wnt5a promotes the maturation of mast cells via the canonical Wnt signaling pathway.

**Figure 4:**
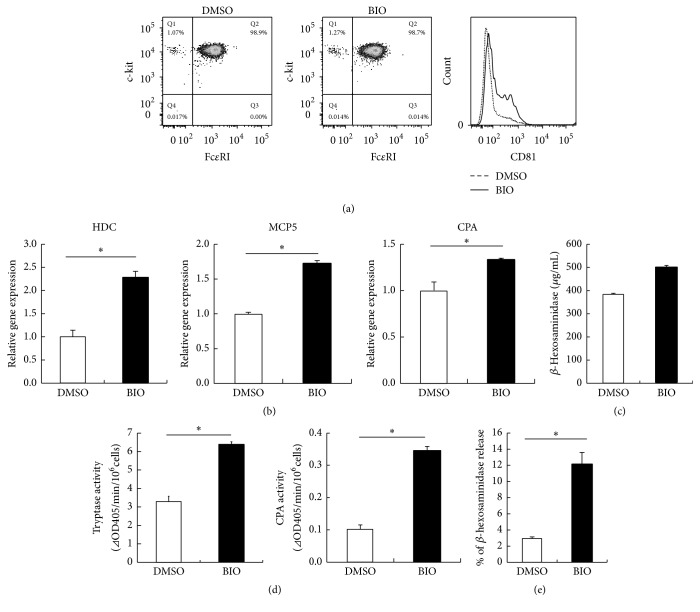
Maturation of mast cells by GSK-3*β* inhibitor. (a)–(e) BMMCs were cultured for 20 days under two conditions: one with IL-3 (1 ng/mL), SCF (100 ng/mL), and BIO and the other with only IL-3 (1 ng/mL) and SCF (100 ng/mL) (control). (a) Suspensions of mast cells were stained with fluorescence-labeled anti-Fc*ε*RI, anti-c-kit, and anti-CD81 antibodies for 30 min on ice. Stained cells were washed, resuspended in 1% FBS-PBS, and analyzed by flow cytometry. Surface marker expression was analyzed by gating on viable cells in the FSC/SSC. One representative dot plot out of five independent experiments is shown. (b) Gene expression levels of HDC, MCP5, and CPA were measured by quantitative RT-PCR. Gene expression levels in the control were taken as 1.0. (c) *β*-Hexosaminidase content was also measured. (d) Granule protease activities of mast cells were measured. (e) *β*-Hexosaminidase release from cells after stimulation with compound 48/80. All data represent the means ± SD (*n* = 4). ^*∗*^ *p* < 0.05.

Next, to study the effect of canonical Wnt signaling pathway on mast cell maturation, BMMCs were treated with an inhibitor of glycogen synthase kinase 3*β* (GSK-3*β*), which regulates the canonical Wnt signaling pathway. 6-Bromoindirubin-3′-oxime (BIO), a GSK-3*β* inhibitor, did not affect the surface expression of either Fc*ε*RI or c-kit on mast cells (Figure 4(a)). FACS analysis showed that expression of CD81 was elevated in the BMMCs treated with BIO (Figure 4(a)). BIO induced the expression of HDC, MCP5, and CPA mRNA (Figure 4(b)). We also found that BIO could induce *β*-hexosaminidase (Figure 4(c)). In addition, the activities of tryptase and CPA in BIO-treated BMMCs were increased as compared with those in the control (Figure 4(d)). Furthermore, the release of *β*-hexosaminidase by compound 48/80 in BIO-treated BMMCs was increased as compared with that in the control (Figure 4(e)). These results showed that the inhibition of GSK-3*β* could promote the maturation of BMMCs into CTMC-like phenotype, suggesting that the canonical Wnt signaling pathway is likely to be involved in the maturation of mast cells.

**Figure 5:**
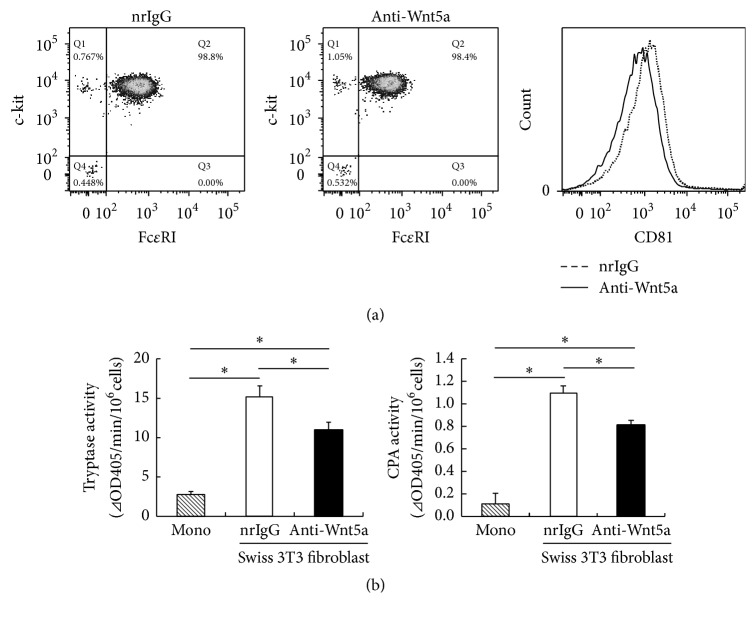
Effect of Wnt5a inhibition on the maturation of mast cells by coculture with Swiss 3T3 fibroblasts. (a)-(b) BMMCs were cultured for 16 days under three conditions: (1) BMMCs were cultured with IL-3 (1 ng/mL) and SCF (100 ng/mL), (2) BMMCs were cocultured with Swiss 3T3 fibroblasts in the presence of SCF (100 ng/mL) and anti-Wnt5a antibody (1 *μ*g/mL), and (3) BMMCs were cocultured with Swiss 3T3 fibroblasts in the presence of SCF (100 ng/mL) and normal rat IgG control (1 *μ*g/mL). (a) Suspensions of mast cells were stained with fluorescence-labeled anti-Fc*ε*RI, anti-c-kit, and anti-CD81 antibodies for 30 min on ice. Stained cells were washed, resuspended in 1% FBS-PBS, and analyzed by flow cytometry. Surface marker expression was analyzed by gating on viable cells in the FSC/SSC. One representative dot plot out of three independent experiments is shown. (b) Granule protease activities of mast cells were measured. All data represent the means ± SD (*n* = 4). ^*∗*^ *p* < 0.05.

Finally, we studied the involvement of endogenous Wnt5a in fibroblast-driven maturation of mast cells using anti-Wnt5a antibody and found that the inhibition of Wnt5a led to a slight decrease in the expression of CD81 (Figure 5(a)). In addition, the activities of tryptase and CPA in anti-Wnt5a antibody-treated BMMCs were slightly decreased as compared with those in the control (Figure 5(b)), suggesting that Wnt5a is not fully responsible for mast cell maturation in this coculture system. Thus, these results suggest that Swiss 3T3 fibroblast-induced maturation of BMMCs to CTMC-like cells is partially mediated by Wnt5a signaling.

## 4. Discussion

Mast cells undergo terminal differentiation in peripheral tissues, although the maturation mechanism of mast cells is poorly understood. Here we report that Wnt5a can induce maturation of mast cells via the canonical Wnt signaling pathway.

In our study on Wnt receptor expression, we found that Fzd4 and LRP5, but not Ror2, were expressed by mast cells. In addition, we found that Wnt5a promoted the maturation of mast cells via the Wnt/*β*-catenin pathway. As previously described, Wnt5a is known to activate *β*-catenin-independent pathway (noncanonical Wnt pathway). On the other hand, Mikels and Nusse recently reported that Wnt5a can also activate the Wnt/*β*-catenin pathway (canonical Wnt pathway) in the presence of the appropriate Wnt receptors, Fzd4 and LRP5, but not in the presence of Ror2 [18]. These results were consistent with our finding that Wnt5a is involved in mast cell maturation. However, the expression of several Fzd mRNAs was detected by RT-PCR analysis (data not shown), suggesting that mast cells may be responsive to instructions from a variety of Wnt signals.

Wnt signaling has been found to regulate the differentiation and function of several blood cell lineages, including hematopoietic progenitors. Scheller et al. showed that constitutive activation of *β*-catenin in the hematopoietic system inhibits the multilineage differentiation of HSCs, resulting in severe anemia, thrombocytopenia, and a decrease in bone marrow cellularity [20]. However, the role of *β*-catenin in the maturation and differentiation of mast cells remains unknown, and further investigations are needed on this subject.

Our results showed that Swiss 3T3 fibroblast-induced maturation of BMMCs to CTMC-like cells would be partially mediated by Wnt5a signaling. Not only the Wnt5a pathway but also other unknown factors are likely to be involved in the Swiss 3T3 fibroblast-promoted maturation of BMMCs, because treatment of anti-Wnt5a antibody did not completely block the maturation of BMMCs. Identification of Swiss 3T3 fibroblast-derived maturation factors would be important to elucidate the mast cell maturation induced by Swiss 3T3 fibroblasts.

Specialized niche environments specify and maintain stem and progenitor cells, but little is known about the functional interactions of niche components with mast cells* in vivo*. In the present study, we demonstrated that Wnt5a promoted the maturation of mast cells. Previous studies have demonstrated that Wnt5a is expressed in hair follicles, which play a role as a reservoir of mast cell progenitor cells [21]. Further studies will be clearly needed to obtain a detailed account of the mechanisms of Wnt-mediated maturation of mast cells in peripheral tissues.

Wnt signaling has critical functions in cellular processes, including differentiation, growth, and apoptosis, and has been studied as a therapeutic target in several disorders, such as type II diabetes, cancer, and Alzheimer's disease. In this study, we demonstrated that Wnt signaling plays an important role in the maturation of mast cells. Previous studies have demonstrated that mast cell progenitors migrate into peripheral tissues and mast cells mature in the tissue environment during allergic pulmonary inflammation in mice [22, 23]. Consequently, Wnt5a may be a potential therapeutic target in allergic diseases.

In summary, we showed that Wnt5a promoted the maturation of mast cells via the canonical Wnt signaling pathway. Our results could facilitate clarification of the mechanisms that control the development of mast cells.

## 5. Conclusions

In this study, we showed that Wnt5a could promote the maturation of mast cells via the canonical Wnt signaling pathway.

## Figures and Tables

**Table 1:** Primer list used in semiquantitative PCR.

Gene name	(5′) Forward primers (3′)	(5′) Reverse primers (3′)
GAPDH	ACCACAGTCCATGCCATCAC	TCCACCACCCTGTTGCTGTA
Fzd4	GCCCAACTTAGTGGGACACG	GGCACATAAACCGAACAAAGGAA
LRP5	AAGGTTGTCGGAACCAACCCATGT	TGATCGTCTTGAGGCTGACATCAGT
Ror1	GGTCAGATCGCTGGTTTCAT	CGTTTGCTTCCTGATTGGAT
Ror2	CCCAACTTCTACCCAGTCCA	TGTCCGCCACAGATGTATTG
Wnt1	GGTTTCTACTACGTTGCTACTGG	GGAATCCGTCAACAGGTTCGT
Wnt2	GCAACACCCTGGACAGAGAT	ACAACGCCAGCTGAAGAGAT
Wnt3	TTCCCATCTCTCCTTGGAGA	CCCGGAAACATGACTTTATCA
Wnt5a	GGACCACATGCAGTACATTGG	CGTCTCTCGGCTGCCTATTT
Wnt6	CTTCGGGGATGAGAAGTCAA	AGCCCATGGCACTTACACTC
Wnt7a	TCGGGAAGGAGCTCAAAGT	GCCACAGTCGCTCAGGTT
Wnt10a	CCTGAACACCCGGCCATAC	GGAGTCTCATTCGAGCATGGAT

**Table 2:** Primer list used in quantitative real-time PCR.

Gene name	(5′) Forward primers (3′)	(5′) Reverse primers (3′)
GAPDH	TTCACCACCATGGAGAAGGC	GGCATGGACTGTGGTCATGA
HDC	CGCTCCATTAAGCTGTGGTTTGTGATTCGG	AGACTGGCTCCTGGCTGCTTGATGATCTTC
MCP5	GGCGGGAGTGTGGTATGC	CCTGGGTTCCAGCACCAA
CPA	GCTACACATTCAAACTGCCTCCT	GAGAGAGCATCCCGTGGCAA
Axin2	TGACTCTCCTTCCAGATCCCA	TGCCCACACTAGGCTGACA
Tcf7	CCAGTGTGCACCCTTCCTAT	AGCCCCACAGAGAAACTGAA
